# An elusive Neotropical giant, *Hondurantemna chespiritoi* gen. n. & sp. n. (Antemninae, Mantidae): a new lineage of mantises exhibiting an ontogenetic change in cryptic strategy

**DOI:** 10.3897/zookeys.680.11162

**Published:** 2017-06-14

**Authors:** Henrique M. Rodrigues, Julio Rivera, Neil Reid, Gavin J. Svenson

**Affiliations:** 1 Department of Invertebrate Zoology, Cleveland Museum of Natural History, 1 Wade Oval Drive, Cleveland, Ohio, USA; 2 Department of Biology, Case Western Reserve University, 10900 Euclid Avenue, Cleveland, Ohio, USA; 3 Universidad San Ignacio de Loyola, Peru; 4 Museo de Entomología Klaus Raven Büller, Universidad Nacional Agraria La Molina, Lima, Peru; 5 School of Biological Sciences, Queen’s University Belfast, MBC, 97 Lisburn Road, Belfast, BT9 7BL, Northern Ireland, UK; 6 Operation Wallacea Ltd., Wallace House, Old Bolingbroke, Lincolnshire, PE23 4EX, England, UK

**Keywords:** Mantodea, Praying mantis, Dictyoptera, crypsis, Mantodea, mantis religiosa, Dictyoptera, crypsis

## Abstract

We present the description of a new genus and new species of praying mantis, *Hondurantemna chespiritoi* **gen. n. & sp. n.** This species of cryptic mantis, collected in National parks in Mexico and Honduras, remained unknown despite its considerable body size. Based on a phylogenetic analysis with molecular data and traditional morphological analysis, we place this new genus within Antemninae, a monotypic Mantidae subfamily. We update the subfamily concept for Antemninae and provide a key to the two genera. We describe the external morphology of immatures and adults of the new species as well as the genital complexes of both sexes and the ootheca of *Antemna rapax*. The observed morphological changes between immature and adult females suggests that the selection for an alternate strategy for crypsis is a response to size increase of the abdomen during development. Immatures exploit a stick/branch habitat based on their morphological appearance while adult females appear as a leaf to disguise the profile of the body.

## Abstract (es)

Se presenta la descripción de un nuevo género y una nueva especie de mantis religiosa, *Hondurantemna chespiritoi* **gen. n. & sp. n**. Esta nueva especie de mantis críptica, colectada en ciertos parques nacionales de México y Honduras, había permanecido hasta ahora desconocida para la ciencia a pesar de su gran tamaño corporal. Utilizando un análisis filogenético con datos moleculares y junto con análisis tradicional de morfología, se logró clasificar a este nuevo género dentro de la subfamilia monotípica Antemninae que pertenece a la familia Mantidae. Como resultado se actualiza el concepto taxonómico de Antemninae y se presenta una clave para identificar los dos géneros atribuidos a esta subfamilia. Se describe la morfología externa de los juveniles y adultos de la nueva especie, así como el complejo genital de ambos sexos y la ooteca de *Antemna rapax*. La diferencia morfológica observada entre juveniles y adultos sugiere que existen fuerzas selectivas divergentes, posiblemente en respuesta al aumento del tamaño del abdomen durante el desarrollo, para así mantener su camuflaje a lo largo de su ciclo de vida. Sobre la base de su apariencia general, los juveniles utilizarían una estrategia críptica asemejando ramas, mientras que las hembras adultas asemejan hojas verdes para ocultar el contorno corporal.

## Introduction

The taxonomy of Mantodea has been revised several times during the 20th century, leading to unstable family and subfamily arrangements (Giglio-Tos 1927, Beier 1964, Terra 1995). Ehrmann and Roy (Ehrmann 2002) proposed the most recent classification of the order, which greatly improved the classification proposed by Beier (1964), although some problems remained. Svenson and Whiting (2004, 2009) conducted molecular phylogenetic analyses and found evidence that around half of the families and subfamilies were not monophyletic, concluding that patterns of convergent morphology mislead taxonomists into creating artificial groups. Their findings indicated the need of revisionary work at almost all levels of Mantodea systematics to better understand the diversity and create a natural classification. In phylogenetic studies, most Neotropical mantises are recovered in two major clades (Yager and Svenson 2008, Svenson and Whiting 2009). The first clade is comprised of the primitively deaf species, the Acanthopoidea (recently revised by Rivera and Svenson 2016), diverging early in the evolution of the group while the second clade diverged much later and includes almost all Neotropical species within Mantidae (Svenson and Whiting 2009). Finally, Rivera (2010b) reviewed the state of the systematics of Neotropical genera and highlighted several groups that needed revision, including all Mantidae subfamilies, calling for increased studies on the diversity and taxonomy of the group.

Two enigmatic praying mantis specimens, one male and one female, were discovered in collections from the United States and France. Shared characteristics of the two specimens suggested they were conspecific, but they were incompatible with the descriptions of known Neotropical genera. Additional material collected in Honduras, including two adult females and nymphs of both sexes, confirmed the conspecificity of the specimens and provided the opportunity to study some morphological variation. Initial examination of other Neotropical taxa allied our unknown specimens with *Antemna rapax* Stål, 1877, the only representative of Antemninae. In both species, mid- and hindlegs present a posteroventral sub-apical lobe on the femora; males have a medial ocellar process; and the shape of the forewing of the females is similar, with an enlarged costal area, which is unusual for Neotropical species. No other Neotropical genus included with the Acanthopoidea *sensu* Rivera and Svenson (2016), Vatinae *sensu* Svenson et al. (2016), Stagmomantinae - Stagmatopterinae - Choeradodinae *sensu* Ehrmann and Roy (Ehrmann 2002) demonstrated enough similarity to suggest close relation. However, the high level of morphological convergence in the order (Svenson and Whiting 2009) prevented a definitive placement of the new lineage in the absence of a phylogenetic analysis. The description of immatures of this new species also presents an opportunity to shed new light on an often-neglected aspect of praying mantis natural history: ontogenetic changes in morphological traits associated with camouflage and mimicry.

Ontogenetic studies on Mantodea are uncommon and most species are described based on adult specimens, while nymphs remain unknown or undescribed, with a few exceptions. Heitzmann-Fontenelle (1969) revised *Cardioptera* Burmeister, 1838 and described the nymphal stages of two species of the genus while Terra (1980) studied the development of raptorial forelegs in four species of Neotropical mantises. Avendaño and Sarmiento (2011) did the most complete work by characterizing morphological changes during the post-embryonic development of *Callibia diana* Stål, 1877 while collecting data to characterize allometric growth. However, these works dealt with cases where the morphology of immatures and adults were similar enough that matching life stages was not difficult. Wieland (2013) reviewed Mantodea post-embryonic development of different body parts and recent studies have revealed a number of cases across Mantodea where nymphal strategy and appearance differ from adults. For instance, nymphs of *Acontista* Saussure & Zehntner, 1894 (Acontistidae) have been reported to resemble ants both in overall shape and in behavior, a strategy not seen in adults (Salazar 2003). The Amazonian *Mantillica nigricans* Westwood, 1889 (Thespidae, Bantiinae) was recently reported to also exhibit ant-mimicry, a strategy observed in nymphs of both sexes as well as adult females, but not in adult males (Agudelo and Rafael 2014). Immature males of the lichen-mimic *Pseudopogonogaster kanjaris* Rivera & Yagui, 2011 (Thespidae, Pseudopogonogastrinae) exhibit cuticular, lichen-like lobes on the abdominal terga similar to those observed in adult females, but in the adult stage these become reduced and tucked under the well-developed wings (Rivera et al. 2011).

Morphological sexual dimorphism, frequently present in Mantodea species (Hurd 1999), can be reflected during development as considerable changes occurring between instars. In cases of moderate to extreme dimorphism, males, females and nymphs have been described as separate genera due to discrepant morphologies, e.g. *Antemna* Stål, 1877, *Phyllomantis* Saussure, 1892 and *Neacromantis* Beier, 1931 (Rehn 1935), *Pseudopogonogaster* Beier, 1942 and *Calopteromantis* Terra, 1982 (Rivera et al. 2011), and *Pachymantis* Saussure, 1871 and *Triaenocorypha* Wood-Mason, 1890 (Svenson et al. 2015). Careful study including adults and immatures can prevent life stages being described as separate taxa or resolve instances where that happened. Studies tracking changes during post-embryonic development not only add descriptive knowledge (e.g. Heitzmann-Fontenelle 1969, Avendaño and Sarmiento 2011) they can also reveal different life strategies between immatures and adults (e.g. Salazar 2003) and shifts between male and female ecologies.

Herein, we leverage a molecular based phylogeny paired with traditional comparative morphology to place our unknown taxon among other Neotropical praying mantises. We 1) place our unknown taxon in a newly created genus allied with *Antemna* within Antemninae; 2) provide a diagnosis for the new genus and an extensive description of the new species, high-resolution images and morphological illustrations; and 3) describe morphological changes occurring through post-embryonic development with comments on the ecological significance and present the distribution for the new species.

## Methods

### Specimens examined and depository

We examined twenty specimens of the new taxon, one male genitalia and one ootheca of *Antemna rapax*. Specimens are accessioned in four collections: the Muséum National d’Histoire Naturelle (MNHN) Paris, France, the California Academy of Sciences (CAS) San Francisco, USA, the Academy of Natural Sciences of Drexel University (ANSP) and the Cleveland Museum of Natural History (CLEV) Cleveland, USA.

### Morphology

We examined specimens using a Leica M80 stereomicroscope and measured them with a Leica M165C stereomicroscope fitted with an IC80 HD coaxial video camera and the live measurement mode of the Leica Application Suite (LAS). Measurements are given in millimeters and include: body length (measured from the frons to the apex of the abdomen); prozona length (measured from the anterior end of the prothorax to the sulcus above the supracoxal dilation); metazona length (measured from the sulcus above the supracoxal dilation to the posterior end of the prothorax); prothorax width (measured at the level of the supracoxal dilation); forewing length (measured from the point where the forewing articulates with the thorax to the tip); hindwing length (measured from the point where the hindwing articulates with the thorax to the tip); forecoxa length (measured from the articulation with the prothorax to the articulation with the trochanter); forecoxa width (measured at the widest point of the coxa); forefemur length (measured from the articulation with the trochanter to the articulation with the tibia); forefemur width (measured at the widest point of the femur); foretibia length (measured from the articulation with the femur to the articulation with the tarsus); mesofemur length (measured from the articulation with the trochanter to the articulation with the tibia); metafemur length (measured from the articulation with the trochanter to the articulation with the tibia). We calculated ratios useful for species identification (Svenson 2014, Rodrigues and Cancello 2016) for the new species: metazona length/prozona length; pronotum length/width; pronotum length/forecoxa length; forefemur length/width. We give spine count for forelegs according to the spination formula proposed by Rivera (2010a) and modified by Brannoch and Svenson (2016), e.g. F= 3DS/10AvS/4PvS; T=12AvS/15PvS where F is forefemur, T is foretibia, DS is discoidal spines, AvS is anteroventral spines and PvS is posteroventral spines. All spines are numbered from the base of the segment to the apex. We extracted male and female genital complexes from the abdomen and treated them in a heated weak KOH solution for 5 minutes to dissolve soft tissues (Rodrigues and Cancello 2016). Treated genital structures were either placed inside vials filled with glycerin and pinned with the specimen or placed with the specimen inside an ethanol-filled vial. Nomenclature of genital structures follows Klass (1997, 1998). Morphological features of the adult female and nymphs that are identical in the males were omitted from the description to avoid redundancy.

### Digital imaging

We took high resolution photos with a Passport Storm^©^ system (Visionary Digital™, 2012), which included a Stackshot z-stepper, a Canon 5D SLR, macro lenses (50mm, 100mm and MP-E 65mm), three Speedlight 580EX II flash units with initial image processing done on Adobe Lightroom 3.6. Z-stepper was controlled using Zerene stacker 1.04, and images stacked with P-Max protocol. Photos were edited using Adobe Photoshop CC to correct for background noise and to add scale bars. We created illustrations with Adobe Illustrator CC based on high-resolution photos of the structures. We constructed plates with Adobe Illustrator CC.

### Phylogenetic analysis

We conducted phylogenetic analyses to test the position of our new taxon using molecular data. The ingroup sample included taxa within Antemninae, Stagmatopterinae, Stagmomantinae, and Vatinae (Table 1), which provided coverage across the four major lineages of Neotropical Mantidae. Representative outgroup taxa were included to test the position of our new taxon relative to Acanthopoidea (Rivera and Svenson 2016) and African and Asian taxa recovered in close relation to our ingroup (Svenson and Whiting 2009). Representatives of Chaeteessidae and Mantoididae were included to root the phylogeny based on their consistent recovery as the two earliest branches of Mantodea (Svenson and Whiting 2009, Wieland 2013, Svenson et al. 2015). We assembled a molecular dataset from previously published works (Svenson and Whiting 2004, 2009, Svenson et al. 2016) and newly generated sequence data (see Table 1 for GenBank accession numbers). The molecular dataset included four genes: the mitochondrial cytochrome oxidase I (COI) and NADH dehydrogenase subunit 4 (ND4) and the nuclear Histone subunits 2 and 3 (H2A and H3). Lab protocols for extraction, amplification, and sequencing followed published procedures (Svenson and Whiting 2004, 2009, Svenson et al. 2015). New sequence data was imported, verified, and aligned along with published data using Geneious alignment on Geneious v7.1.4. The resulting alignment included 3512 characters. We determined the best fit models for each gene using the Akaike Information Criterion implemented in MEGA v.7 (Kumar et al. 2015): GTR+Ɣ+I for COI, T92+Ɣ for ND4, HKY+Ɣ for H2A and T92+I for H3. We conducted four independent mixed model Bayesian inference (BI) using MrBayes ver. 3.2.5 (Altekar et al. 2004, Ronquist et al. 2012). For all BI, each run was started from a random tree. All sampled generations (every 1000) prior to stationarity were discarded (burn-in). The trees sampled from the stationary distribution were summarized as a 50% majority rule consensus tree to find posterior probabilities (PP) (Huelsenbeck and Imennov 2002, Huelsenbeck et al. 2002). We also performed partitioned maximum likelihood (ML) analysis using RAxML v8 (Stamatakis 2014). Nucleotide substitution parameters were estimated independently from each data partition. One thousand nonparametric bootstrap (BS) pseudoreplicates were performed under a GTR model with CAT approximation of Gamma-distributed among-site rate heterogeneity. Every fifth BS tree was used as a starting tree for more thorough optimization of the real data under GTR+Gamma. FigTree v1.4.2 (Rambaut 2012) was used to visualize topologies and produce figures for both ML and Bayesian analyses.

**Table 1.** List of species used in the phylogenetic analyses with their families and subfamilies given.

Species	Family	Subfamily	MN Code	GenBank accession number – COI	GenBank accession number – H2A	GenBank accession number – H3	GenBank accession number – ND4
*Mantoida* sp.	Mantoididae	-	MN180	FJ802822.1	-	FJ806771.1	FJ802586.1
*Chaeteessa* sp.	Chaeteessidae	-	MN482	KR360623.1	-	KR360691.1	-
*Acanthops falcataria*	Acanthopidae	Acanthopinae	MN112	EF383861.1	-	EF384117.1	FJ802526.1
*Macromantis nicaraguae*	Mantidae	Photininae	MN144	EF383873.1	-	EF384129.1	FJ802554.1
*Cardioptera squalodon*	Mantidae	Photininae	MN178	EF383889.1	-	EF384145.1	FJ802584.1
*Epaphrodita musarum*	Acanthopidae*	Epaphroditinae	MN435	KY783773	-	KY783805	KY783826
*Hymenopus coronatus*	Hymenopodidae	Hymenopodinae	MN010	EF383800.1	KR360626.1	AY491334.1	FJ802425.1
*Choeradodis rhombicollis*	Mantidae	Choeradodinae	MN016	EF383805.1	-	AY491340.1	FJ802431.1
*Rhombodera basalis*	Mantidae	Mantinae	MN344	FJ802913.1	-	FJ806867.1	FJ802738.1
*Vates pectinicornis*	Mantidae	Vatinae	MN014	EF383803.1		AY491338.1	FJ802429.1
*Vates* sp.	Mantidae	Vatinae	MN760	-	-	KY783825	-
*Zoolea orba*	Mantidae	Vatinae	MN351	KT732082.1	-	KT732101.1	-
*Oxyopsis* 1	Mantidae	Stagmatopterinae	MN294	FJ802868.1	-	FJ806822.1	FJ802689.1
*Oxyopsis* 2	Mantidae	Stagmatopterinae	MN734	KY783777	-	KY783808	KY783828
*Oxyopsis* 3	Mantidae	Stagmatopterinae	MN739	KY783780	-	KY783811	KY783829
*Oxyopsis* 4	Mantidae	Stagmatopterinae	MN743	KY783784	-	KY783815	KY783832
*Oxyopsis* 5	Mantidae	Stagmatopterinae	MN749	-	-	-	KY783841
*Parastagmatoptera sottilei* 1	Mantidae	Stagmatopterinae	MN740	KY783781	-	KY783812	-
*Parastagmatoptera sottilei* 2	Mantidae	Stagmatopterinae	MN742	KY783783	KY783797	KY783814	KY783831
*Parastagmatoptera flavoguttata* 1	Mantidae	Stagmatopterinae	MN741	KY783782	KY783796	KY783813	KY783830
*Parastagmatoptera flavoguttata* 2	Mantidae	Stagmatopterinae	MN751	KY783788	KY783801	KY783819	KY783836
*Parastagmatoptera vitreola* 1	Mantidae	Stagmatopterinae	MN731	KY783775	KY783794	KY783806	-
*Parastagmatoptera vitreola* 2	Mantidae	Stagmatopterinae	MN735	KY783778	-	KY783809	-
*Stagmatoptera hyaloptera*	Mantidae	Stagmatopterinae	MN733	KY783776	-	KY783807	KY783827
*Stagmatoptera septentrionalis*	Mantidae	Stagmatopterinae	MN029	FJ802763.1	-	AY491353.1	FJ802444.1
*Stagmatoptera supplicaria* 1	Mantidae	Stagmatopterinae	MN117	EF383863.1	-	EF384119.1	FJ802531.1
*Stagmatoptera supplicaria* 2	Mantidae	Stagmatopterinae	MN736	KY783779	KY783795	KY783810	-
*Stagmatoptera supplicaria* 3	Mantidae	Stagmatopterinae	MN748	KY783787	KY783800	KY783818	KY783835
*Stagmomantis* 1	Mantidae	Stagmomantinae	MN747	-	KY783804	KY783824	-
*Stagmomantis* 2	Mantidae	Stagmomantinae	MN744	KY783785	KY783798	KY783816	KY783833
*Stagmomantis* 3	Mantidae	Stagmomantinae	MN745	KY783786	KY783799	KY783817	KY783834
*Stagmomantis* 4	Mantidae	Stagmomantinae	MN730	KY783774	KY783793	-	-
*Stagmomantis* 5	Mantidae	Stagmomantinae	MN753	KY783789	KY783802	KY783820	KY783837
*Antemna rapax*	Mantidae	Antemninae	MN147	EF383875.1	-	EF384132.1	FJ802557.1
*Hondurantemna chespiritoi* gen. n. & sp. n. 1	Mantidae	Antemninae	MN757	KY783790	-	KY783821	KY783838
*Hondurantemna chespiritoi* gen. n. & sp. n. 2	Mantidae	Antemninae	MN758	KY783791	-	KY783822	KY783839
*Hondurantemna chespiritoi* gen. n. & sp. n. 3	Mantidae	Antemninae	MN759	KY783792	KY783803	KY783823	KY783840

*Follow the classification of Terra, 1995 although the correct placement of the genus is currently unknown.

## Results

### Phylogeny

**Figure 1.**
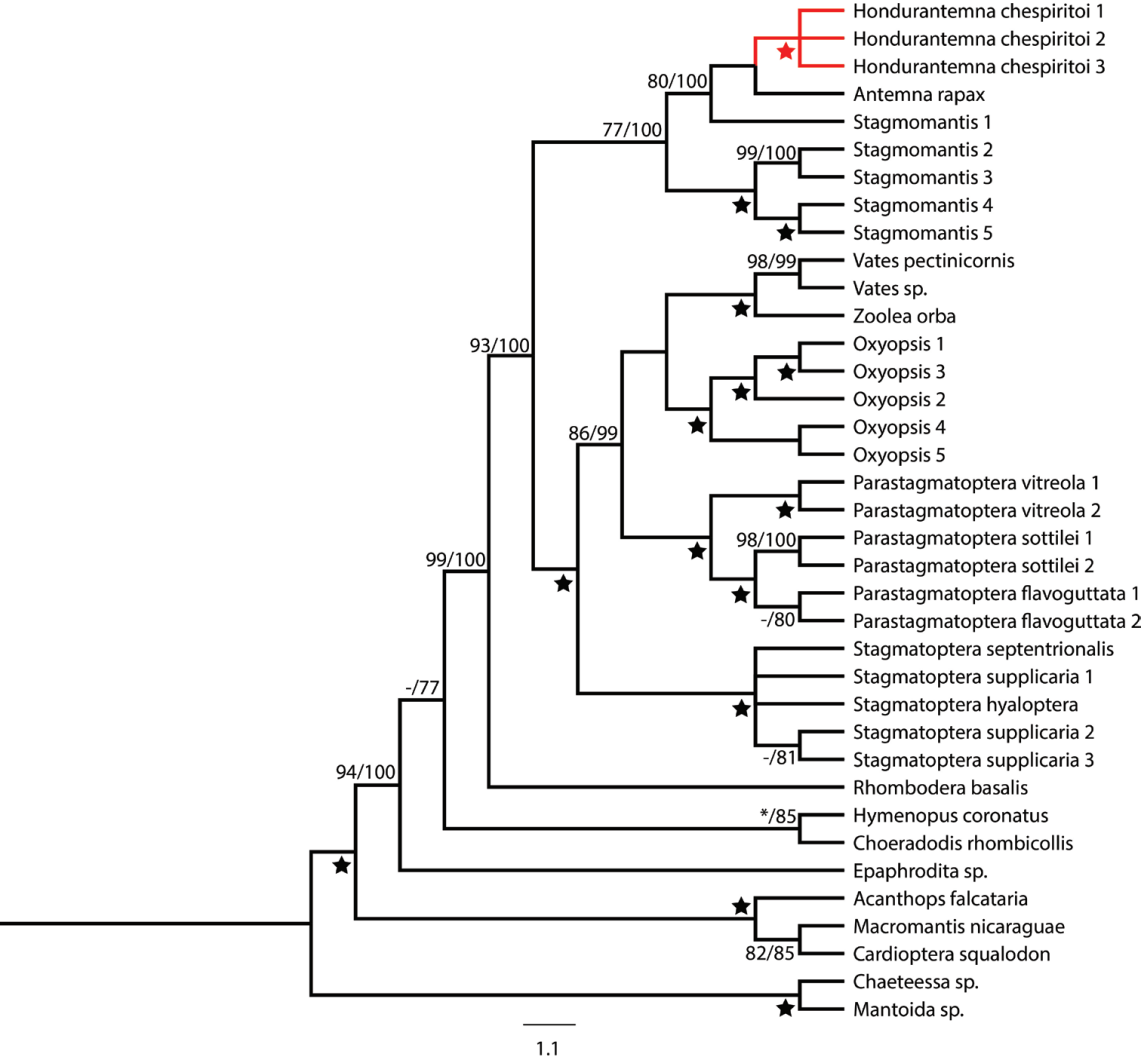
Bayesian Inference (BI) tree with bootstrap values from the Maximum-likelihood and posterior probabilities values higher than 70 shown. Star represent clades with maximum support in both analyses, dash represents support lower than 70 in one of the analyses and asterisk represents a clade recovered only in the BI analysis. *Hondurantemna chespiritoi* n.gen. n. sp. is highlighted in red.

The results of BI and ML analyses were largely congruent and generally well supported (Fig. 1). The partitioned ML analysis recovered a topology (likelihood score: -22681.630120) with a moderate BS value (83) resolving our new taxon as sister to *Antemna* within a clade including one *Stagmomantis* Saussure, 1869 taxon (Fig. 1). However, BS values within (*Stagmomantis* 1 + *Antemna* + the new taxon) indicate taxon relationships within the clade and with other *Stagmomantis* taxa may be unstable. The BI (harmonic mean: -22653.69) also recovered our new taxon as sister to *Antemna* with high PP, with the remainder of the topology in almost complete congruence with the ML topology. PP for nodes within the (*Stagmomantis* 1 + *Antemna* + the new taxon) clade firmly place the new taxon within this lineage (Fig. 1). We recovered *Stagmomantis* 1 as sister to the new genus plus *Antemna* in both analyses rather than sister to other *Stagmomantis* taxa, indicating a paraphyletic *Stagmomantis*.

### Systematics

The phylogenetic analyses recovered the new taxon as closely related to *Antemna rapax* (Fig. 1). This result corroborates our initial hypothesis based on morphological observations and supports inclusion of the new taxon within Antemninae. Terra (1995) created the subfamily Antemninae to accommodate only the type-genus *Antemna*. With the description of a new genus within Antemninae, we update the concept of the subfamily to reflect its new composition. We also present the first photos of the male genitalia of *Antemna rapax* (see below) (Figs 20, 21).

#### Antemninae

Taxon classificationAnimaliaMantodeaMantidae

Terra, 1995

##### Type-genus.

*Antemna* Stål, 1877.

##### Diagnosis.

Males have a medial ocellar process that originates posteriorly to the ocelli but anterior to the postfrontal sulcus. The dorsal margin of the forefemora at least partially produced, forming a lamellar projection. Forefemora with four discoidal spines. Meso- and metathoracic legs bearing a posteroventral sub-apical lobe originating from an expansion of the keel running along the margin of the femur. Abdomen of the females swollen, almost as wide as long.

##### Key to Antemninae genera

**Table:** 

1	Five posteroventral forefemoral spines. Dorsal expansion of forefemur extending to femoral apex (Fig. 7). Forewing of females with a spot on the center of the discoidal area. Apofisi falloide of male genitalia with the anterior apex recurved dorsally (Fig. 12). Processo ventrale of the right phallomere longer than wide (Fig. 13)	***Hondurantemna* gen. n.**
–	Four posteroventral forefemoral spines. Dorsal expansion of forefemur ending abruptly before femoral apex (Fig. 19). Forewing of females with a spot on the border of the discoidal and costal areas. Apofisi falloide of male genitalia with the anterior apex not recurved dorsally (Fig. 20). Processo ventrale of the right phallomere wider than long (Fig. 21)	***Antemna***

#### Hondurantemna gen. n.

Taxon classificationAnimaliaMantodeaMantidae

http://zoobank.org/4B298C0B-9D51-44ED-9E12-860116CA0E24

Figs 2 , 3 , 4 , 5 , 6 , 7 , 8 , 9 , 10 , 11 , 12 , 13 , 14 , 15 , 16 , 17 , 18

##### Type species.

*Hondurantemna chespiritoi* sp. n. by monotypy

##### Diagnosis.

Rounded compound eyes, nymphs and adult males have a medial ocellar process, subadult and adult females without the process. Forelegs with five posteroventral spines and four discoidal spines. Mid- and hindlegs with a single small lobe near the apex of the femur. Male’s forewings are hyaline with green crossveins, forewings of females with a spot close to the center of the discoidal area.

##### Etymology.

The generic epithet combines the words Honduras, country were the majority of specimens we studied were collected, and *Antemna*, in reference to the morphological similarities of both Antemninae genera.

#### Hondurantemna chespiritoi sp. n.

Taxon classificationAnimaliaMantodeaMantidae

http://zoobank.org/08043DE8-6E08-4930-BC22-288FA7C6386B

##### Type-specimens.

**Holotype**. 1 ♂ Mexico, Chiapas, Municipio de La Trinitaria, Lagunas de Monte Bello National Park, bellow Dos Lagos on rd. to Santa Elena, 1219m, 14.x.1981, D.E. & P.M. Breedlove (CAS). **Allotype**. 1 ♀ Honduras, Cortes 18km O. San Pedro Sula, Cra. El Merendon, 1650m, vii.1995, T. Porion A. Grange (MNHN). **Paratypes**. *Adults*: 1♀ Honduras, Cortez, San Pedro Sula, Cusuco National Park, Base Camp, 15.4964 -88.2119, 27.vii.2015, N. Reid col. GD0073, MN758; 1♀ Honduras, Cortez, San Pedro Sula, Cusuco National Park, Base Camp, 15.4964 -88.2119, 31.vii.2015, N. Reid col. GD0072. *Immatures*: 1♀ Honduras, Cortez, San Pedro Sula, Cusuco National Park, Santo Tomas, 15.5611 -88.2974, 21.vii.2015, N. Reid col.; 1♂ Honduras, Cortez, San Pedro Sula, Capuca, 15.5031 -88.2222, 17.vi.2015, N. Reid col.; 1♂ 6♀ Honduras, Cortez, San Pedro Sula, Cusuco National Park, Base Camp, low vegetation, 15.4964 -88.2119, 09.vi.2015, N. Reid col.; 1♂ Honduras, Cortez, San Pedro Sula, Cusuco National Park, Base Camp, low vegetation, 15.4964 -88.2119, 09.vi.2015, N. Reid col., MN757; 2♀ Honduras, Cortez, San Pedro Sula, Cusuco National Park, Base Camp, 15.4964 -88.2119, 17.vi.2015, N. Reid col.; 1♀Honduras, Cortez, San Pedro Sula, Cusuco National Park, Base Camp, 15.4964 -88.2119, 17.vi.2015, N. Reid col., MN759; 1♀ Honduras, Cortez, San Pedro Sula, Cusuco National Park, Base Camp, 15.4964 -88.2119, 26.vii.2015, N. Reid col.; 2♀ Honduras, Cortez, San Pedro Sula, Cusuco National Park, Base Camp, 15.4964 -88.2119, vi–vii.2015, N. Reid col. (all paratypes are accessioned at CLEV).

##### Description.

**Figure 2.**
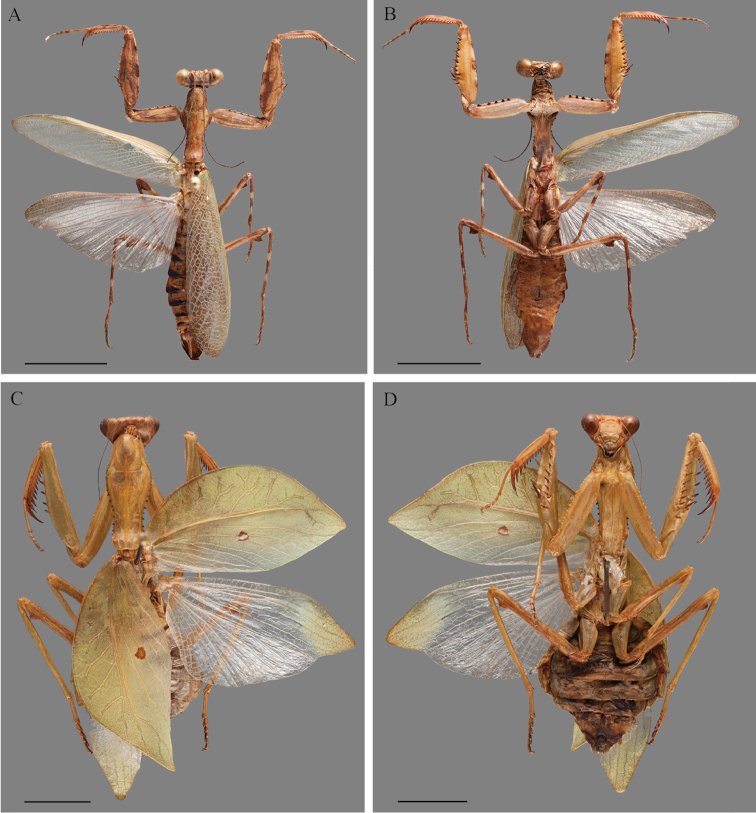
Dorsal and ventral habitus of *Hondurantemna chespiritoi* gen. n. sp. n., **A** dorsal habitus of the male **B** ventral habitus of the male **C** dorsal habitus of the female **D** ventral habitus of the female. Scale bar = 10mm.

***Male***: Medium sized. General coloration light brown with dark brown spots (Fig. 2A, B). Body length: 34.2; prozona length: 2.9; metazona length: 5.9; prothorax width: 3.8; forewing length: 21.9; hindwing length: 18.1; forecoxa length: 7.2; forecoxa width: 1.9; forefemur length: 8.8; forefemur width: 2.4; foretibia length: 5.0; mesofemur length: 12.0; midleg metatarsus length: 1.8; metafemur length: 15.3; hindleg metatarsus length: 2.5; metazona length/prozona length: 2.0; pronotum length/width: 2.3; pronotum length/forecoxa length: 1.2; forefemur length/width: 3.6.

**Figure 3.**
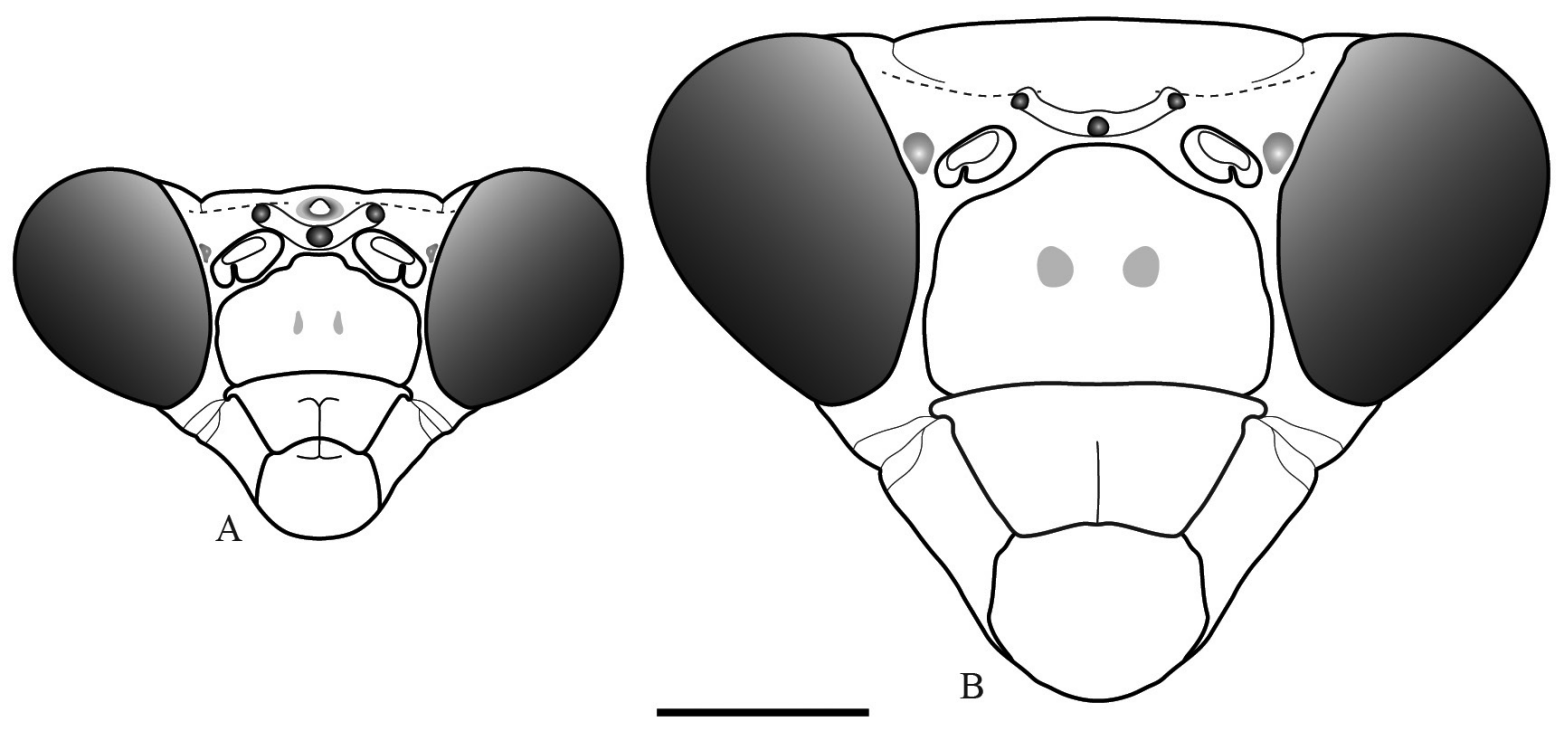
Frontal view of the head of *Hondurantemna chespiritoi* gen. n. sp. n. **A** male **B** female. Scale bar = 2mm.

*Head* (Fig. 3A): Rounded eyes. Vertex with dark brown spots, straight, on the same level as the imaginary line connecting the dorsal margin of compound eyes, and separated from the ocelli by two lateral keels and a central depression, the latter bearing the medial ocellar process with a rounded apex. Juxtaocular bulges not developed, on the same level as the vertex. Ocelli medium sized, arranged in the shape of a “V”, with the two lateral ocelli further away from each other due to the central depression. Scape and pedicel light brown, flagellomeres of antennae filiform, dark brown. One small tubercle between the eye and the antennal socket. Lower frons subpentagonal, almost as high as wide, bearing two small tubercles, upper margin arcuate, sinuous, the apex straight. Clypeus with a vertical central keel on the lower half. Maxillary palps with black spots on the medial surface, progressively larger towards the apical segments. Labial palps black on the medial surface of all segments.

**Figure 4.**
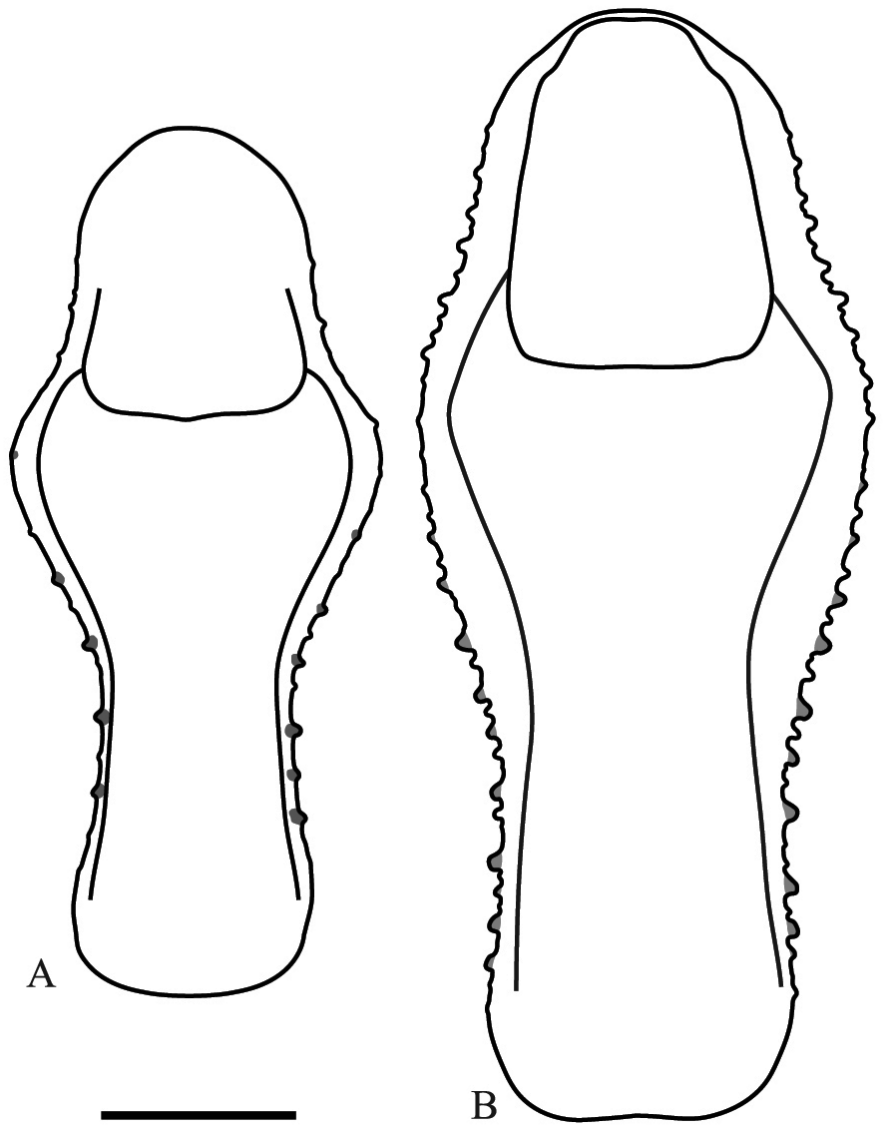
Dorsal view of the prothorax of *Hondurantemna chespiritoi* gen. n. sp. n. **A** male **B** female. Scale bar = 5mm.

**Figure 5.**
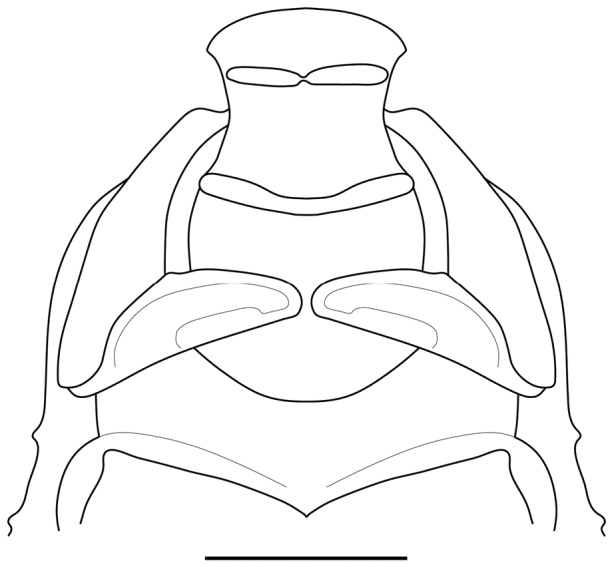
Ventral view of the cervical region of male *Hondurantemna chespiritoi* gen. n. sp. n. Scale bar = 1mm.

*Thorax* (Fig. 4A): General shape cruciform, with the supracoxal dilation pronounced and rounded, metazona two times longer than the prozona. Margins of the prozona convergent anteriorly, ciliated, posteriorly slightly expanded, produced as flat projections, herein called shelves. Margins of the metazona also slightly produced in shelves, ciliated, with small tubercles, almost all of which are black, the anterior part of the metazona with symmetrical depressions on the dorsal surface. Cervix framed by lateral and intercervical sclerites, two ventral sclerites present; the intercervical sclerites with a pronounced torus intercervicalis, the first ventral cervical sclerite constricted in the middle (Fig. 5). Metathoracic hearing organ with deep groove, without knobs (DNK type) (see Yager and Svenson, 2008).

**Figure 6.**
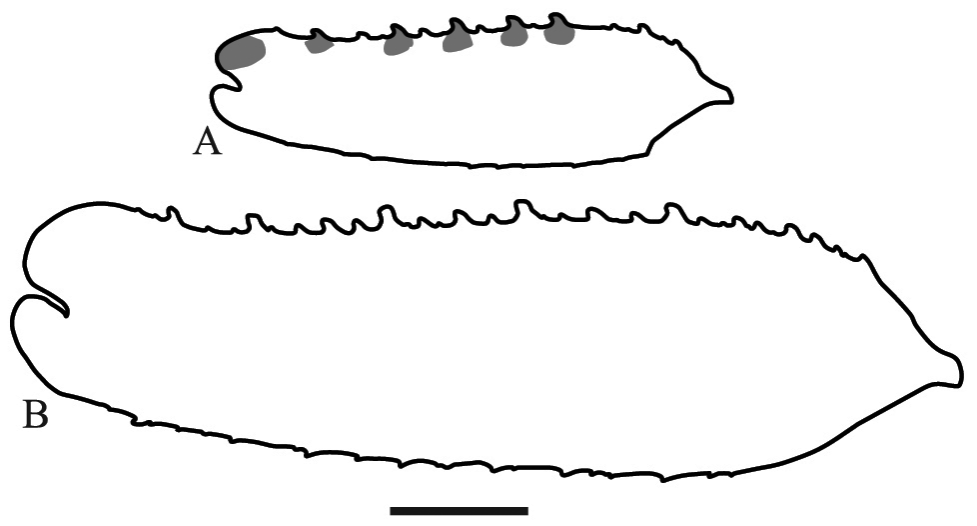
Anterior view of the forecoxa of *Hondurantemna chespiritoi* gen. n. sp. n. **A** male **B** female. Scale bar = 2mm.

**Figure 7.**
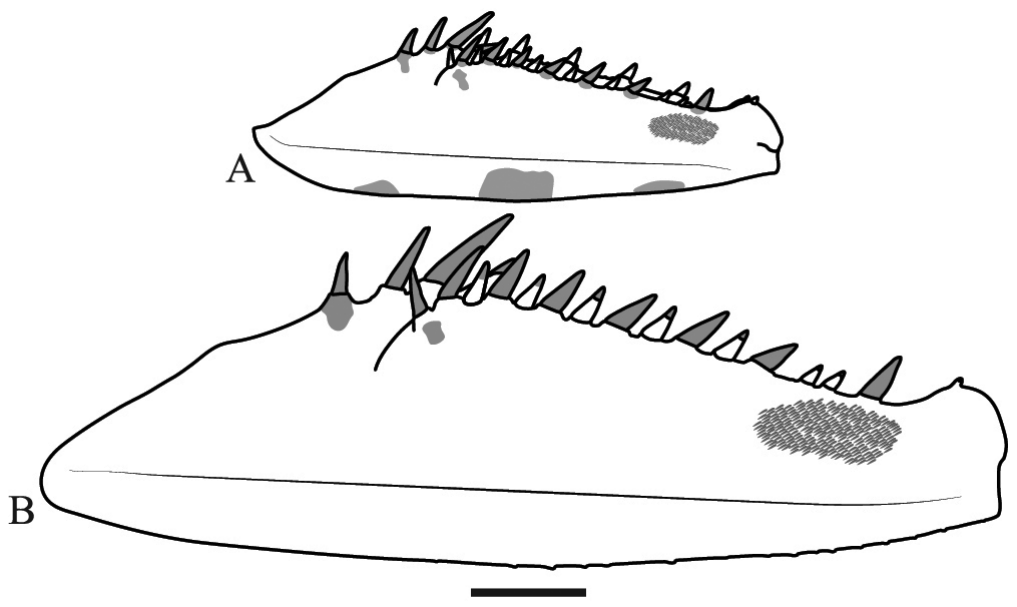
Anterior view of the forefemur of *Hondurantemna chespiritoi* gen. n. sp. n. **A** male **B** female. Scale bar = 2mm.

**Figure 8.**
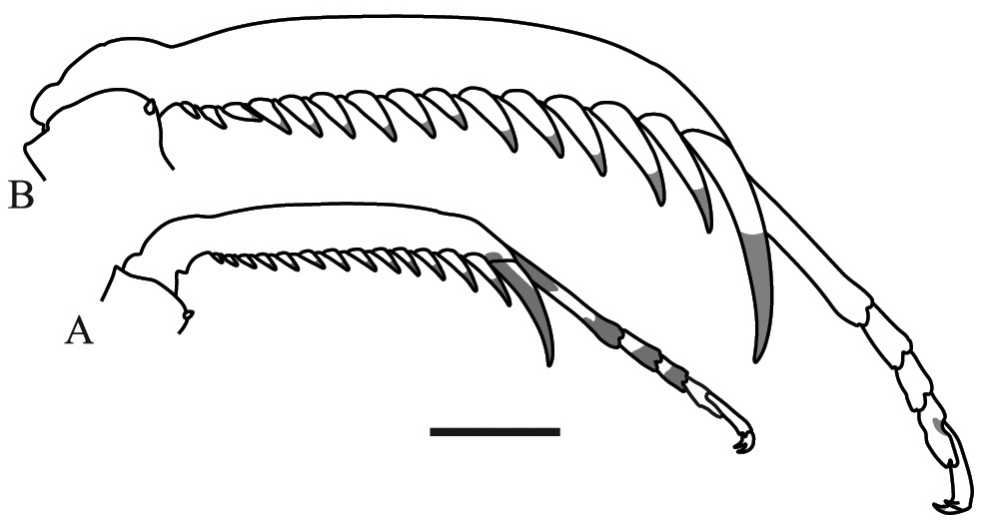
Anterior view of the foretibia of *Hondurantemna chespiritoi* gen. n. sp. n. **A** male **B** female. Scale bar = 2mm.

*Prothoracic legs*: Forecoxae (Fig. 6A) triangular in cross-section, light brown except for the dorsal apical lobe, which is dark brown; posteroventral margin with dispersed tubercles, anteroventral margin with small tubercles bearing slender setae, dorsal margin bearing five large spines and smaller spines between them, the former dark brown on the posterior surface and black on the anterior surface and around the base; anterior apical lobes divergent. Forefemora (Fig. 7A) light brown with three dark brown spots on the dorsal area of the anterior surface, dorsal margin regularly convex, slightly compressed anteroposteriorly; F=4DS/15AvS/5PvS; crenulation between posteroventral spines II, III and IV; all discoidal spines black on the anterior surface, the first spine with a dark spot on its base; all the large anteroventral spines black on the inner surface with a dark spot on their bases, a dark spot on the anterior surface above the first two spines; genicular spine developed on both sides of the femora; spur sulcus located in the proximal quarter of the femora; femoral brush extending from the 13^th^ anteroventral spine to beyond the most distal. Foretibiae (Fig. 8A) light brown; T= 12–13AvS/12–13PvS. Foretarsi light brown, with an anterior-basal dark brown spot on the first tarsomere and dark brown anterior-apical spots on tarsomeres I–III.

*Wings*: Forewings reaching the apex of the abdomen, costal area with a sinuous margin, reticulate veins, opaque green; discoidal area mostly hyaline, with an anterior area smoky green and all the veins and crossveins opaque green. Hindwings shorter than the forewings, not reaching the apex of the abdomen; hyaline with brown-red veins and crossveins (Fig. 2A, B).

**Figure 9.**
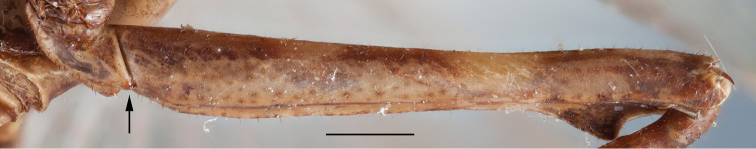
Anterior view of the mesotrochanter and mesofemur of an adult male, arrow showing the spine on the articulation between the trochanter and the femur. Scale bar = 1mm.

**Figure 10.**
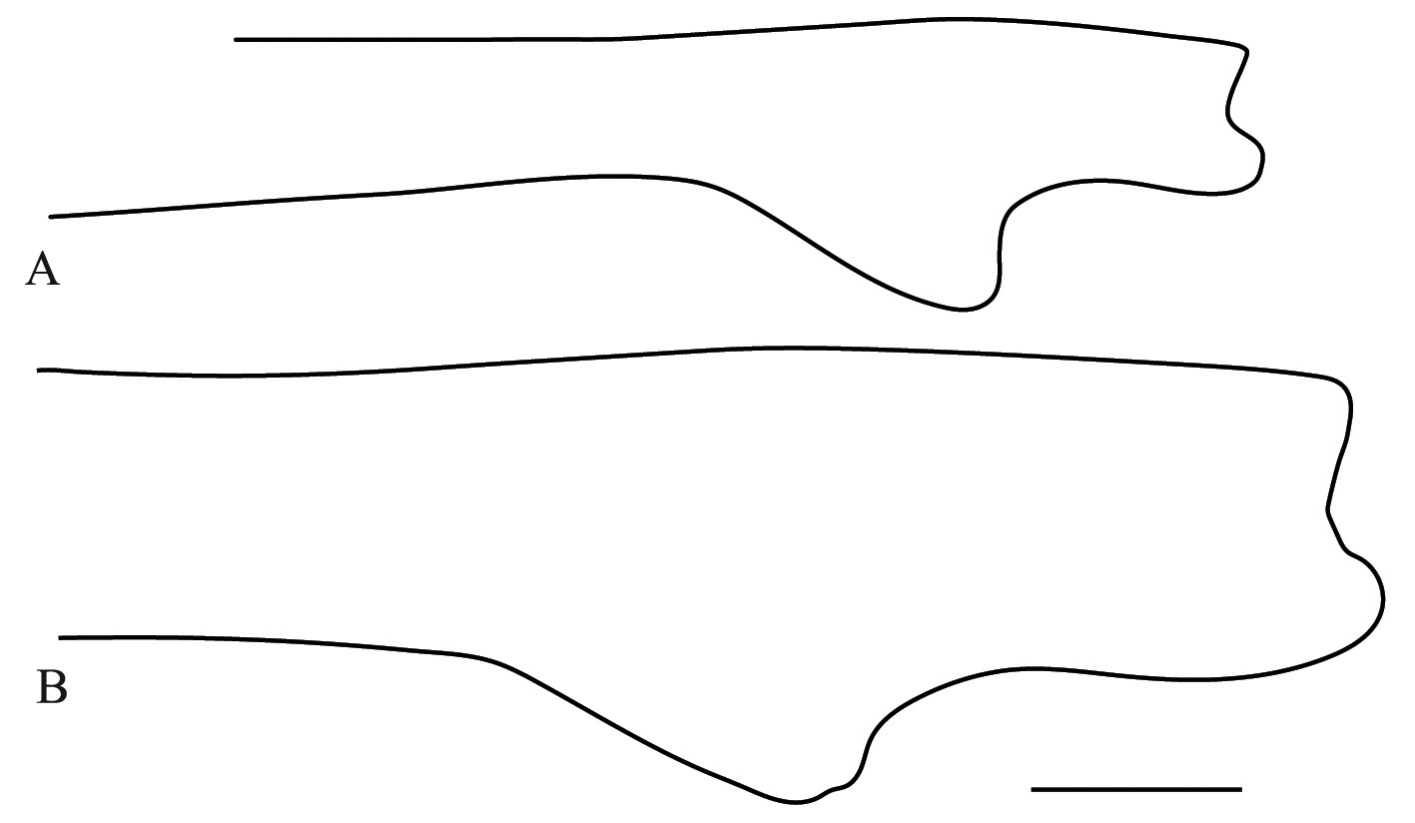
Apex of the hindfemur of *Hondurantemna chespiritoi* gen. n. sp. n. **A** male **B** female. Scale bar = 1mm.

*Meso- and metathoracic legs*: Coxa with two strong keels, one anterodorsal and the other posterodorsal. Trochanter with a notch on apical ventral margin, bearing a spine on the articulation with the femora (Fig. 9). Femora smooth except for one keel that runs along the posteroventral margin and originates one single subapical lobe (Fig. 10A); one genicular spine present on the anterior surface. Tibiae smooth, circular in cross-section with two genicular spines. Tarsi with metatarsomeres shorter than other tarsomeres together.

**Figure 11.**
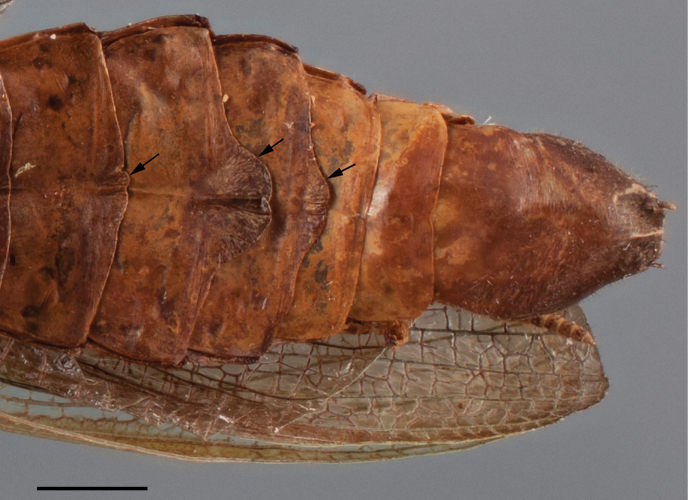
Close up of the ventral side of the abdomen of an adult male of *Hondurantemna chespiritoi* gen. n. sp. n., arrows indicate the ventral lobes. Scale bar = 2mm.

*Abdomen*: With black spots on the sides of tergites II–V and VII–IX, and a black stripe on tergites VI–VII. Slightly dorsoventrally compressed, with apical lobes on sternites IV–VI, more developed on segments V and VI, the lobes flat against the body (Fig. 11). Supra-anal plate triangular, wider than long, posterior margin arcuate. Cerci elongated but not reaching the apex of subgenital plate, the latter almost as wide as long, flat between styli.

**Figure 12.**
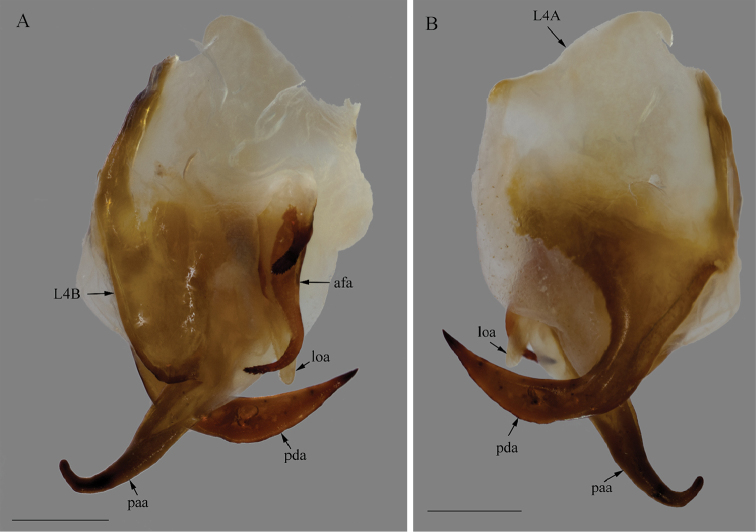
Left phallomere complex of the male genitalia of *Hondurantemna chespiritoi* gen. n. sp. n., afa (apofisi falloide), loa (lobo membranoso), paa (processo apicale), pda (processo distale). **A** dorsal view **B**- ventral view. Scale bar = 1mm.

**Figure 13.**
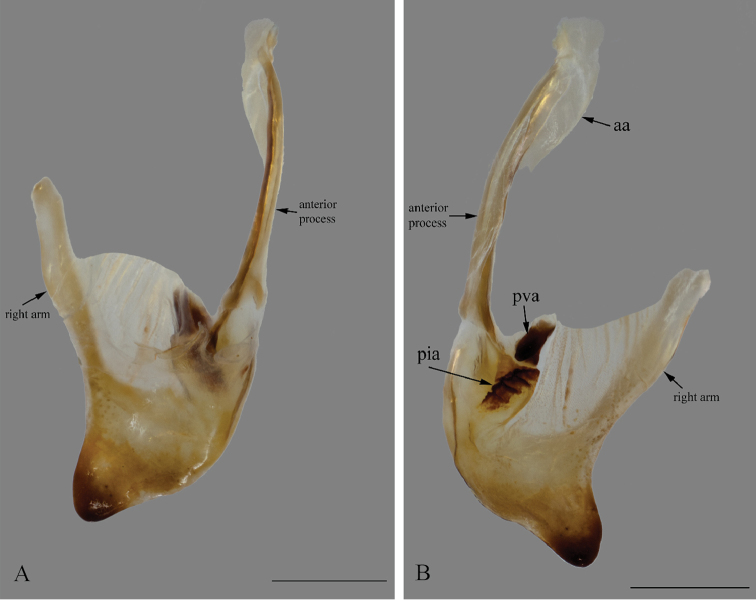
Right phallomere of the male genitalia of *Hondurantemna chespiritoi* gen. n. sp. n., aa (apodema anterior), pia (piastra ventrale), pva (processo ventrale). **A** dorsal view **B** ventral view. Scale bar = 1mm.

*Genitalia*: Left phallomere – Sclerite L4B roughly rectangular, longer than wide, left margin projected anteriorly; apofisi falloide (afa) with the anterior part bearing small spines and extremely elongate, projecting dorso-posteriorly, posterior part also elongated, curved towards the left, tapering distally and bearing small spines at the apex; lobo membranoso (loa) elongate, without projections, glabrous; processo apical (paa) elongate, slightly flattened, curved 30° to the left, apex curved anteriorly; sclerite L4A roughly circular, without projections other than the processo distal (pda); pda elongate, curved to the right, uniformly broad, tapering at the distal third, ending in a strongly sclerotized spine (Fig. 12). Right phallomere – roughly triangular, rounded posterior apex; right arm elongate, slender, without projections; anterior process elongate and slender; apodema anterior (aa) oval and slender; processo ventrale (pva) elongate, smooth, apex rounded and well sclerotized; piastra ventrale (pia) elongate, well sclerotized, with “U” shaped striations (Fig. 13).

***Female***: Medium to large sized. General coloration light green without any spots (Fig. 2C, 2D). Body length: 52.9–53.9; prozona length: 5.1–5.5; metazona length: 10.8–11.2; prothorax width: 6.7–7.0; forewing length: 29.4–31.6; hindwing length: 31.6–31.9; forecoxa length: 12.7–14.0; forecoxa width: 3.0–3.9; forefemur length: 15.6–16.0; forefemur width: 4.3–4.6; foretibia length: 8.5–8.9; mesofemur length: 12.0–12.4; metafemur: length 15.3–15.8; metazona length/prozona length: 2.0–2.1; pronotum length/width: 2.4–2.5 pronotum length/forecoxa length: 1.2–1.3; forefemur length/width: 3.4–3.6.

*Head* (Fig. 3B): Vertex straight or slightly sinuous, raised above imaginary line connecting dorsal margins of the eyes, juxtaocular bulges slightly developed. Ocelli small, arranged in the shape of an arc. Scape, pedicel and first half of the flagellomeres of the antennae green, the second half of the flagellomeres dark brown. Lower frons bearing two small central tubercles, except in one of the specimens. Maxillary palps green, the last segment with black spot on the medial surface.

*Thorax* (Fig. 4B): Supracoxal dilation pronounced and rounded. Margins of the prozona convergent, ciliated with small tubercles, produced in shelves. The posterior third of the metazona with a central keel. The first ventral cervical sclerite in one of the specimens constricted in the middle, in the other two specimens not constricted.

*Prothoracic legs*: Forecoxae (Fig. 6B) green, dorsal margin bearing six to eight large spines, dark brown on the anterior surface and around the base, apical lobes parallel. Forefemora (Fig. 7B) green, dorsal margin bearing small tubercles, regularly convex, slightly compressed anteroposteriorly; F=4DS/15AvS/5PvS; posteroventral spines with crenulation present after the second spine; the first discoidal spine black on the anterior surface, the other three dark brown; the first anteroventral spine and all the large anteroventral spines dark brown on the anterior surface, the large spines may present a dark spot on their base. Foretibiae (Fig. 8B) green; T= 14AvS/13–14PvS. Foretarsi green, without dark spots.

*Wings*: Forewings opaque green, costal area almost as wide as the discoidal area, the apex constricted, making the tegmina follow the abdomen contour, with 7–10 branches of the sub-costa vein, crossveins with a reticulate appearance; spot in the center of the discoidal area, composed of an anterior small crescent-shaped brown portion and a posterior round white portion; anal area smoky green. Hindwings as long as the tegmina, reaching the apex of the abdomen, apex of the discoidal area well developed, opaque green, the remainder of the hindwing hyaline (Fig. 2C, D).

*Meso- and metathoracic legs*: Femora with three keels, one runs along the posteroventral margin and originates one single subapical lobe (Fig. 10B), the second runs along the dorsoposterior margin and the third, less marked, runs along the dorsoanterior margin. Tibiae with two rows of aligned setae. Metatarsi with the metatarsomeres equal or slightly smaller than the other tarsomeres together.

*Abdomen*: Without black spots. Slightly dorsoventrally compressed, without apical lobes on sternites. Cerci elongated, cercomeres cylindrical, except the last one, which is conical.

**Figure 14.**
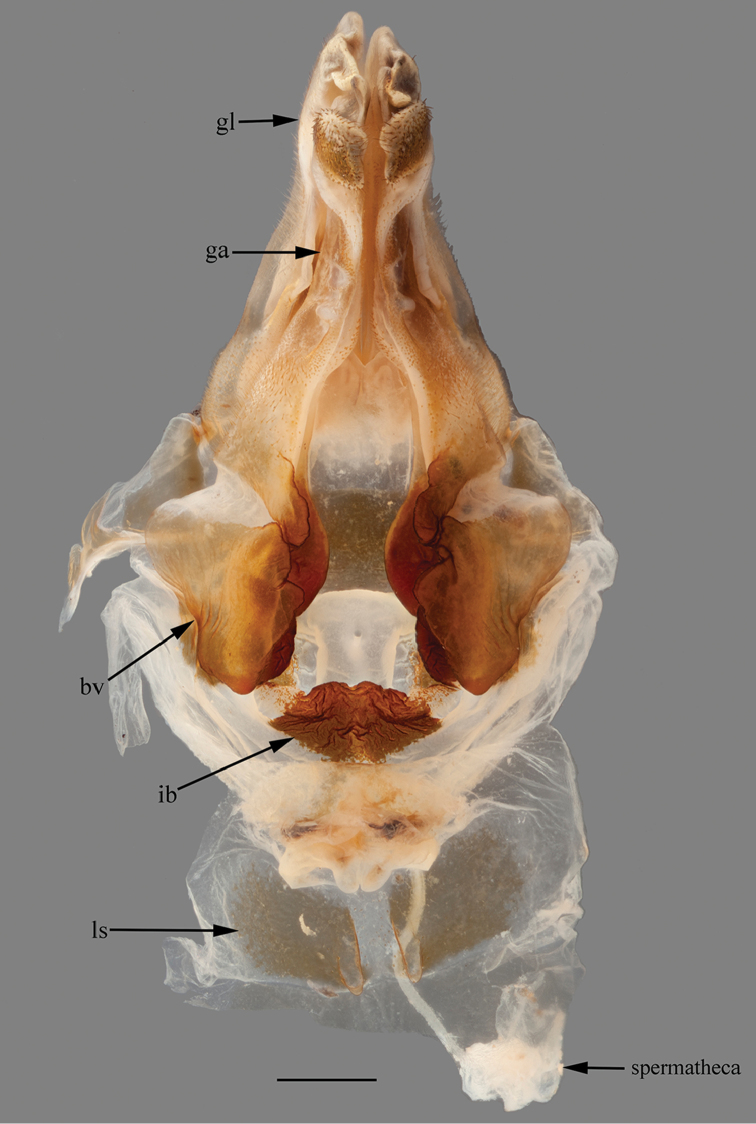
Female genital complex of *Hondurantemna chespiritoi* gen. n. sp. n., bv (basivalvula), ga (gonapophysis VIII), gp (gonoplacs), ib (interbasivalvula), ls (laterosternal shelf). Scale bar = 1mm.

*Genitalia*: Gonoplacs (gl) simple, bearing setae along the dorsal margin and the base, apex bearing a ventral projection. Gonapophysis IX (gp) mostly membranous, with two sclerotized ribbons, one elongate and tapering towards the apex of gp, the other shorter and occupying a medial projection of gp, this projection being rounded; gl and gp of almost the same length. Gonapophysis VIII (ga) bearing setae on the base, on the ventral surface and on the apex, a dorsal groove spanning the two basal thirds, ending in a pointed projection, the apex enlarged ventrally. Basivalvula (bv) with the lateral surface smooth, bearing a central depression, the medial surface rugose, with two projections, one central directed medially, the other more posterior, directed to the base of the ga. Interbasivalvula (ib) well sclerotized, rugose and shaped like a sectioned rhombus. Laterosternal shelves (ls) weakly sclerotized, roughly rhomboid, with short rounded posterior projections (Fig. 14).

***Nymphs*** (unless specified, description applies to male and female nymphs of all instars): General coloration varies from entirely light brown to light brown mottled with dark brown.

**Figure 15.**
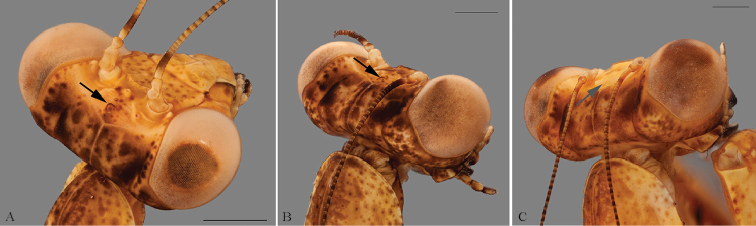
Dorsolateral view of the head of female nymphs of *Hondurantemna chespiritoi* gen. n. sp. n. Black arrows point the medial ocellar process that degenerates during ontogenetic development, grey arrow points the region where the process stood. **A** early instar nymph **B** mid instar nymph **C** late instar nymph. Scale bars = 1mm.

*Head*: Vertex higher than imaginary line connecting dorsal margin of the compound eyes, without lateral keels and a central depression but with the medial ocellar process. In female nymphs, the process becomes increasingly smaller during development, being absent on the instar before the final molt (Fig. 15). Juxtaocular bulges slightly developed. Ocelli small, underdeveloped. Lower frons mottled dark brown to completely light brown, with the upper margin not sinuous, tubercles light brown. Clypeus with a transversal keel on the middle, light brown or with the upper half mottled dark brown and the lower half light brown. Maxillary palps with segments I and II black on the medial surface, IV light brown to light brown with a dark brown spot, segment V light brown to completely dark brown. Labial palps segments black on the medial surface or completely dark brown.

*Thorax*: The first ventral cervical sclerite may be split in two. Metathoracic hearing organ underdeveloped.

*Prothoracic legs*: Forecoxae light brown except for the lower apical lobe, which is dark brown. Dorsal margin bearing five to six large spines. Forefemora varying, light brown with three dark brown spots on the dorsal area of the anterior surface to dark brown with light brown spots; F=4DS/15AvS/5PvS; males without crenulation between posteroventral spines, females with crenulation; discoidal spines black on the anterior surface, the first spine presenting a dark spot on its base; the first anteroventral spine black, a dark spot on the anterior surface above the first two to four spines; femoral brush extending from the 12^th^ anteroventral spine to the last, a dark spot may be present under the femoral brush. T=13–14AvS/12–14PvS. Foretarsi with an anterior-basal dark brown spot on the first tarsomere and dark brown anterior-apical spots on all tarsomeres.

**Figure 16.**
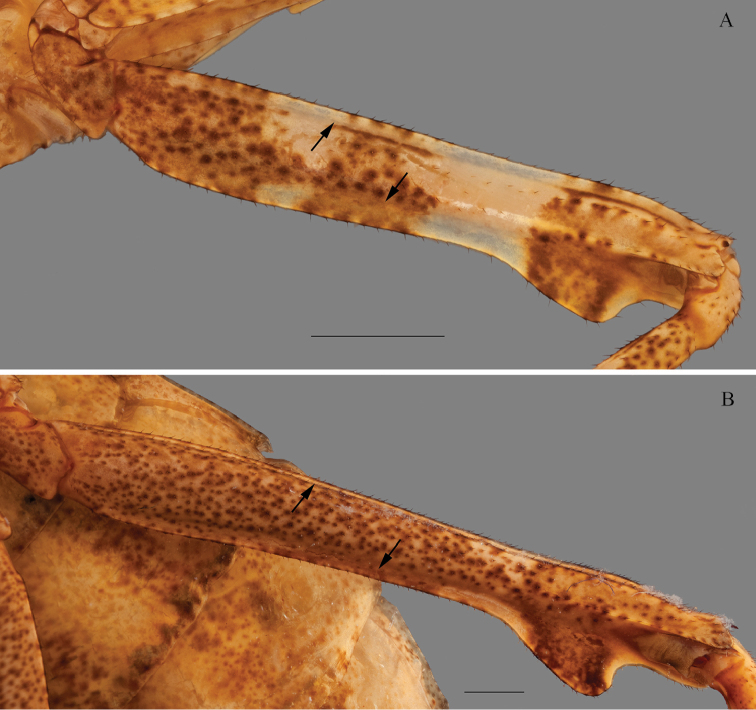
Anteroventral view of the femur of female nymphs of *Hondurantemna chespiritoi* gen. n. sp. n. with arrows showing the end of the expansion of the carina. **A** early instar nymph **B** late instar nymph. Scale bars = 1mm.

*Meso- and metathoracic legs*: Femora with three keels, one runs along the posteroventral margin and originates one single subapical lobe, the second runs along the dorsoposterior margin and the third, less marked, runs along the dorsoanterior margin, the keels on the youngest female nymphs are expanded into shelves (Fig. 16A). Metatarsi with the metatarsomeres equal or slightly smaller than the other tarsomeres together.

**Figure 17.**
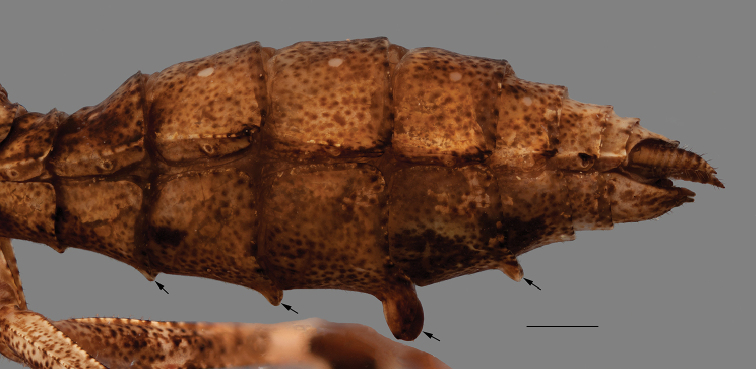
Lateral view of the abdomen of a male nymph of *Hondurantemna chespiritoi* gen. n. sp. n., arrows indicate the ventral lobes. Scale bar = 1mm.

*Abdomen*: Without black spots or bands on the tergites. Not dorsoventrally compressed, on males apical lobes present on sternites IV–VI, lobe on segment V more developed, the lobes pointing down instead of being held against the body (Fig. 17), on females, lobes absent. Supra-anal plate triangular, almost as long as wide, posterior margin arcuate. Cerci short, styli developed on males, absent on females.

**Figure 18.**
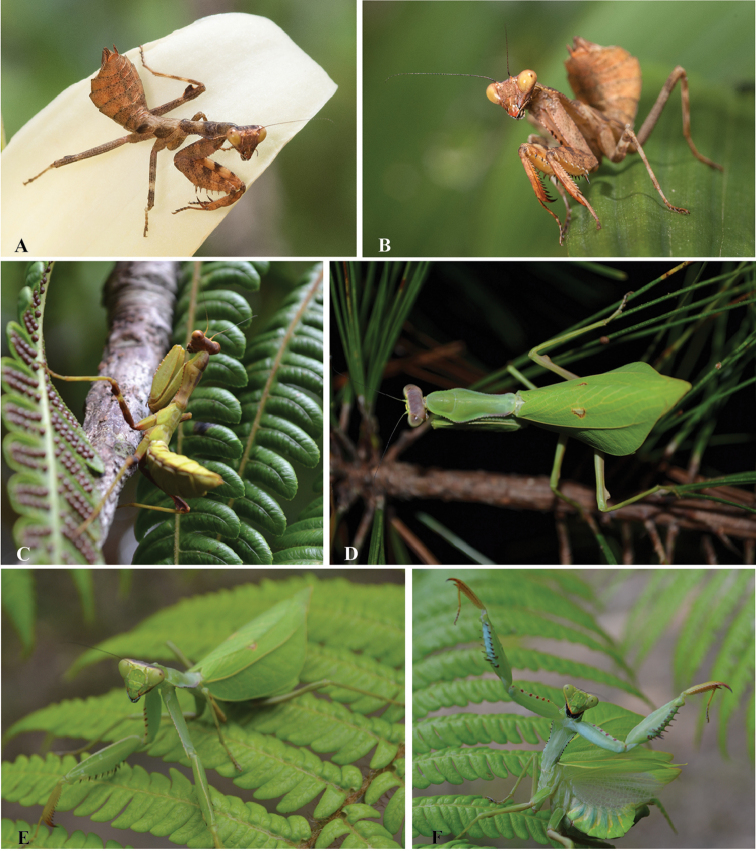
Living specimens of *Hondurantemna chespiritoi*. **A–C** nymph females **D–F** adult females. Female in F is showing deimatic display. Credits: **A–B** by Andrew Snyder, **C–F** by Ethan Staats.

##### Distribution and habitat.

The Lagunas de Montebello National Park, Mexico, from which the holotype was collected, is ca. 6,500 hectares located on the high plains of Chiapas, with an altitude between 1,200 and 1,800 meters above sea level (UNESCO: Lagunas de Montebello http://www.unesco.org/mabdb/br/brdir/directory/biores.asp?mode=gen&code=MEX+37). The predominant vegetation is Central American pine-oak forest over a highly rugged terrain. The Sierra del Merendón, from which the allotype and paratypes were collected, is a mountain range extending from northwest Honduras into southeast Guatemala bordered by the Lempa and Motagua River valleys. The size of the region and its typographical complexity supports four principal forest ecotypes; 1) tropical lowland dry forest, 2) tropical moist forest, 3) montane cloud forest (above 1,200m) and 4) the Bosque Enano or ‘dwarf forest’ occurring at the highest elevations (above 2,000m). The Parque Nacional Cusuco, surrounding Montaña San Ildefonso (also known as Cerro Jilinco), from which the paratypes were collected, is located within the Sierra del Merendón, and is a protected area of 23,440 hectares (Slater et al. 2011). The vegetation is mostly montane secondary broad-leaved forest interspersed with pines, which dominate steeper slopes with palms or bamboo thickets along elevated ridges and tree ferns at lower altitudes (NR, pers. comm.). All nymph specimens were collected in small clearings on low vegetation, usually in the early morning when individuals were found commonly at the apex of herbaceous plants ca. 1-2m tall often hanging upside-down on the underside of a leaf or branch.

##### Etymology.

A name in the genitive case, this species is named after “Chespirito”, the screen name of famous late Mexican TV comedian Roberto Gomez Bolaños. Chespirito created and portrayed several characters cherished across Latin America, including “El Chavo del Ocho” and “El Chapulín Colorado”, the latter a sort of superhero whose outfit was inspired by grasshoppers or “chapulines”.

#### Antemna rapax

Taxon classificationAnimaliaMantodeaMantidae

Stal, 1877

**Figure 19.**
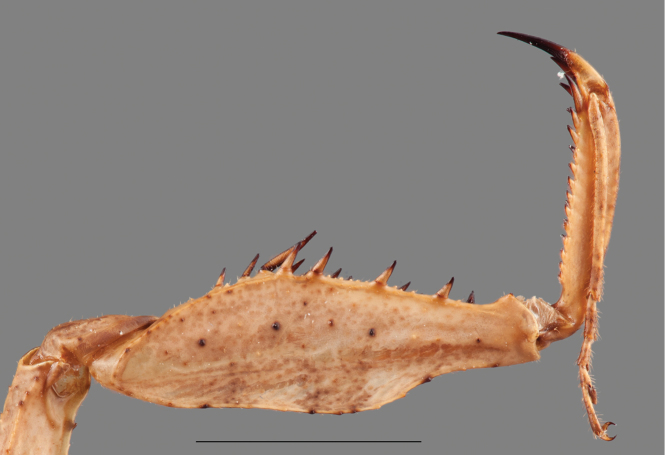
Posterior view of the forefemur of a male *Antemna rapax*. Scale bar = 5mm.

**Figure 20.**
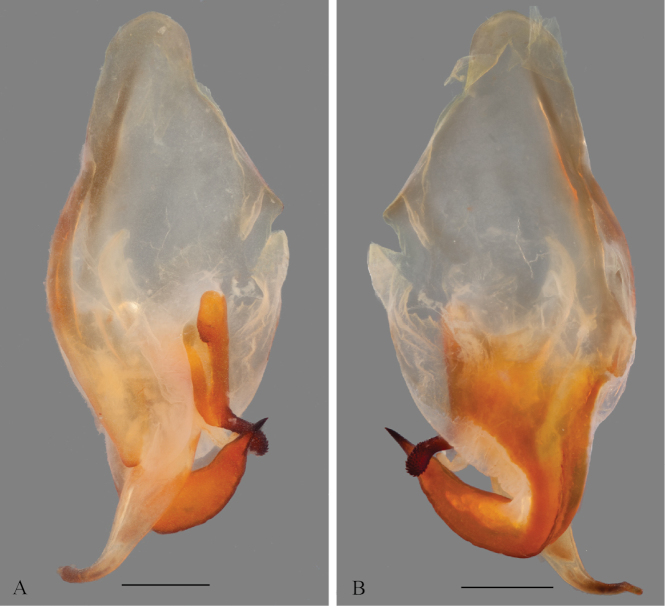
Left phallomere complex of the male genitalia of *Antemna rapax*. **A** dorsal view **B** ventral view. Scale bar = 1mm.

**Figure 21.**
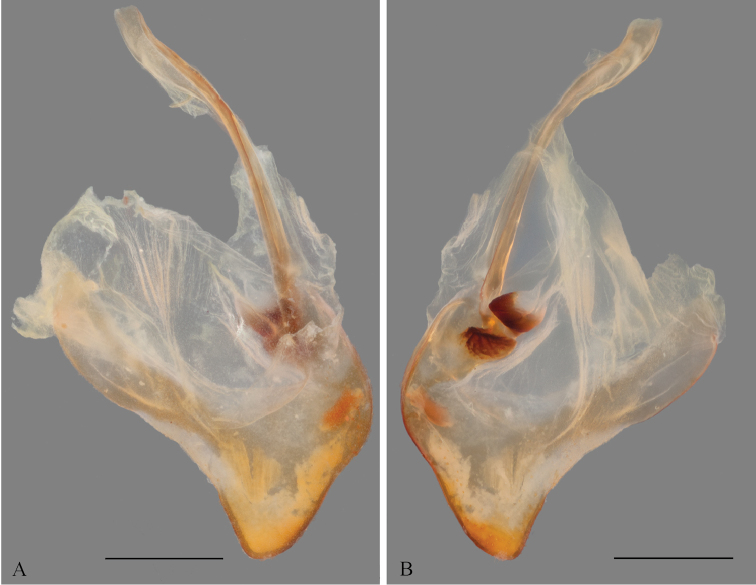
Right phallomere of the male genitalia of *Antemna rapax*. **A** dorsal view **B** ventral view. Scale bar = 1mm.

**Figure 22.**
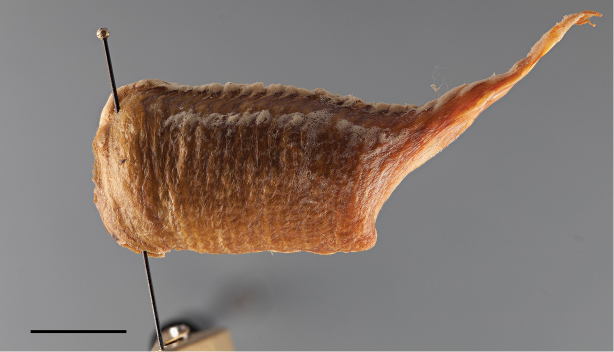
Lateral habitus of the egg case of *Antemna rapax*. Scale bar = 10mm.

Figs 19 , 20 , 21 , 22

##### Description.

*Genitalia*: Left phallomere – Sclerite L4B roughly oval, much longer than wide, left margin projected anteriorly; afa with the anterior part smooth and short, posterior part elongated, curved towards the right, clubbed and bearing spines at the apex; loa elongate, without projections, glabrous; paa elongate, slightly flattened, curved 30° to the left, apex curved anteriorly; sclerite L4A elongate, without projections other than the pda; pda elongate, curved to the right, uniformly broad, tapering at the apex, ending in a sclerotized spine (Fig. 20). Right phallomere – roughly triangular, rounded posterior apex; right arm elongate, broad, without projections; anterior process elongate and slender; aa oval and slender; pva short, smooth, apex blunt and well sclerotized; pia short, well sclerotized (Fig. 21).

Egg case: somewhat barrel-like, laterally compressed, posteroventral end encircling substrate to which it is attached, ventral surface away from, and forming an angle with the substrate. External wall russet brown in color and rough in appearance. External coating in the form of a whitish layer of frothy material. The coating extends over the emergence area and adjacent dorsal surface of egg case. Exhibiting 40–44 egg chambers whose boundaries are clearly visible as sigmoidal markings along the dorsolateral surface the egg case (external coating might conceal this feature in some specimens), and with as many emergence openings as egg cambers, aligned to form two parallel rows along the dorsal margin. Some emergence openings can be seen on a long, relatively straight, and upwardly projected, distal process (Fig. 22). Approximated measurements: length: 35; width: 15; distal process: 22.

##### Examined material.

Male genitalia: Costa Rica, Puntarenas, P. N. Piedras Blancas, Esquinas Lodge (NW – Golfito), 15.v.1996, D. Brzoska, GSMC005332, GD0097 (CLEV). Ootheca: *Antemna rapax*, ANSP.

## Discussion

Our molecular phylogenetic analyses confirmed our morphology based hypothesis of a unique taxon, *Hondurantemna chespiritoi*, being placed within the subfamily Antemninae. Our analysis also recovered Antemninae and Stagmomantinae as closely related, with the latter recovered as paraphyletic. This result suggests that current classification does not represent natural lineages, a pattern that repeats across various major lineages within Mantodea (Svenson and Whiting 2009, Svenson et al. 2015, Rivera and Svenson 2016). Our results being incongruent with current classification is due to either: 1) Antemninae is a lineage within Stagmomantinae, or 2) *Stagmomantis* does not represent a monophyletic genus and Stagmomantinae is likely in need of revision. Given the highly complex taxonomic history, and poorly understood relationships within *Stagmomantis* (Maxwell 2014), it is likely that the genus in its current composition is not monophyletic. We recovered a nymphal specimen, listed as *Stagmomantis* 1, as sister to (*Antemna* + *Hondurantemna*) (Fig. 1). *Stagmomantis* 1 was collected in the Dominican Republic and this specimen is likely *Stagmomantis domingensis* (Palisot de Beauvois, 1805), a species placed in a monospecific genus, *Isomantis* Giglio-Tos, 1917, which Terra (1995) synonymized with *Stagmomantis*. The position of *Stagmomantis* 1 in this analysis indicates that it is not a true *Stagmomantis*, adding support to Giglio-Tos’ (1917) *Isomantis*. However, a larger taxon and gene sampling within Stagmomantinae is necessary to better understand the organization within Stagmomantinae and its affinities with other lineages.

There are two non-exclusive explanations for differing morphologies in immatures and adults. Adaptive responses to changes in habitat and resource (i.e. prey) use during postembryonic development, e.g. *Acontista* (Salazar 2003) or different selective pressures on adult males and females leading to sexual dimorphism (Slatkin 1984, Shine 1989, Svenson et al. 2016). Praying mantis females are thought to be under selective pressure for increased fitness leading to a more cryptic life style, while males are selected for increased mobility and mate location (Edmunds 1976, Hurd 1999). As the development of immatures progresses, morphological differences between males and females become more accentuated, with nymphs resembling only one of the sexes, e.g. *Pseudopogonogaster* (Rivera et al. 2011).

Nymphs of both sexes and adult males of *Hondurantemna chespiritoi* exhibit cryptic features to resemble small twigs. These include brown coloration, presence of a medial ocellar process (Figs 3A and 15A), mid- and hindlegs bearing lobes (Figs 9, 10, 16), and the presence of abdominal, sternal lobes on males (Figs 11 and 17). All of these characters can have a masquerading effect by disrupting the body outline, making difficult location of individuals in their natural environment (Edmunds and Brunner 1999). In females, as immatures reach adulthood, the medial ocellar process becomes increasingly small and eventually is lost (Fig. 15), the size of the leg lobes relative to the leg decreases (Fig. 16), coloration changes from brown to green (Fig. 18), and the abdomen swells. This latter feature is particularly pronounced, as the abdomen becomes increasingly enlarged during postembryonic development due to the production of large egg masses (Roy 1999). The enlarged abdomen would be prohibitive to a twig-like cryptic strategy in females, as body outline becomes too distinct and conspicuous. We hypothesize that different selective pressures on females forced their departure from the twig strategy employed as a nymph and drove the reduction of twig-like characters and the evolution of enlarged, leaf-like forewings that could conceal the large abdomen. Their predominately green coloration would additionally enhance the cryptic effect by blending within strongly green vegetation. These features would help adult females resemble leaves as their central cryptic strategy, unlike adult males, which retain the same cryptic strategy of nymphs.

The ontogenetic changes of *Hondurantemna chespiritoi* brings to question the identity of nymphs described by Agudelo et al. (2002). The authors described first instar nymphs, which they assigned to Antemninae based on the morphological similarity of the nymphs with adults of *Antemna rapax*. Those nymphs, despite their general similarity to *A. rapax*, lacked the medial ocellar process and femoral lobes on the mid- and hindlegs, characteristic of *A. rapax*. The authors noted those characters could develop during posterior development, but chose not to assign the nymphs to a genus until early instar nymphs of *A. rapax* or the adult of the nymphs they described became known. The changes during ontogenetic development of *Hondurantemna* described here are comparable to the changes expected if those nymphs are *A. rapax*. However, the *Antemna* ootheca described here is distinct from the description and images provided by Agudelo et al. (2002). Additional material and studies are necessary to confirm the placement of those nymphs within Neotropical Mantodea.

## Conclusion

*Hondurantemna chespiritoi* is exemplary of large, undiscovered insect diversity yet to be documented. Although *H. chespiritoi* has fascinating post-embryonic changes that indicate distinct selective pressures acting on both sexes, the pattern may be more common than we realize, considering the lack of information on nymphal biology. This study shows the importance of describing all life stages and both sexes whenever possible, to prevent misidentification of conspecific specimens and bring to light the natural history of the species.

## Supplementary Material

XML Treatment for Antemninae

XML Treatment for Hondurantemna

XML Treatment for Hondurantemna chespiritoi

XML Treatment for Antemna rapax
